# Surgical stabilization of rib fracture patients versus nonoperative controls treated by a multidisciplinary team in a single institution

**DOI:** 10.1016/j.heliyon.2023.e15205

**Published:** 2023-04-07

**Authors:** Frank Bauer, Susan Haag, Kaveh Najafi, Brian Miller, John Kepros

**Affiliations:** Surgical and Critical Care, HonorHealth Scottsdale Osborn Medical Center, Scottsdale, AZ, 85251, USA

**Keywords:** Rib fixation, Rib fracture, Surgical stabilization of rib fracture, Multidisciplinary care

## Abstract

**Introduction:**

Despite promising evidence, surgical stabilization of rib fractures (SSRF) is not ubiquitously offered in all trauma centers. Some centers struggle with patient selection while some struggle due to surgeon comfort with the technique. To address this issue, our trauma center developed a multidisciplinary SSRF approach between orthopedic and trauma surgery.

**Methods:**

This retrospective study compared 43 patients who underwent SSRF at a level 1 trauma center with 43 nonoperatively managed controls. Our study Indications were flail chest with >3 segments; non-flail with severe, bi-cortical displacement of >3 contiguous segments. Main outcome measures included mortality, ICU duration, hospital stay LOS, rates of ventilator-associated pneumonia (VAP) and ventilator days.

**Results:**

Results of SSRF included decreases in mortality (2% vs 16.3%; *p* = 0.03) and in ICU duration. Patients with SSRF had a significantly shorter duration in the ICU than the nonoperative group (8.72 vs 14 days; *p* = 0.013) but a similar hospital duration (LOS mean, 12.81 vs 15.2; *p* = 0.29). Less patients in the SSRF group developed VAP but the difference was not significant (2% vs 14%, ***p* = 0.055**).

**Discussion:**

SSRF patient outcomes supported prior evidence. The tandem approach had benefits as surgeons were able to leverage skills and expertise, increase collaboration between services, and complete more difficult reconstructions. Our experience may serve as a model for trauma centers interested in starting a new program or enhancing current service offerings.

## 1 Introduction

Rib fractures are seen in 10% of all trauma admissions and up to 39% of patients after thoracic trauma [1,2]. Chest wall injury is a common in those presenting with high impact trauma due to falls, which may contribute to increased mortality and complications such as increased hospital admission and intensive care unit (ICU) duration [3]. In addition, rib fractures can be associated with high morbidity with pulmonary conditions that include pneumothorax and hemothorax [4]. Thus, some require mechanical ventilation. The majority of patients who require mechanical ventilation are treated non-operatively with pain management and discharged with few complications [5]. Yet, some patients undergo surgical fixation [6].

The gold standard of rib fracture management has been pain control, pulmonary hygiene, and mechanical ventilation [7]. Yet, increasing evidence supports surgical stabilization of rib fractures (10.13039/100009666SSRF) as an effective and safe intervention [7,8]. Stabilization of the chest wall, particularly when a flail segment is present, has been associated with reduced rates of pneumonia, number of days in the ICU, and in mortality [4,9,10]. Cohort studies and meta-analyses have revealed positive outcomes [7,[11], [12], [13], [14], [15], [16], [17], [18], [19], [20]].

Surgical stabilization of rib fractures is not ubiquitously offered in all trauma centers [21]. While the number of those receiving SSRF has increased in recent years, the number remains small. One study [22] used the 2016 National Trauma Data Bank (NTDB), which had 114,972 total patients with rib fractures meeting inclusion criteria; of those, a total of 113,306 (98.6%) were treated nonoperatively and 1666 (1.4%) were treated with SSRF. Another study estimated only 5.8% of patients with flail chest (FC) undergo SSRF [23].

Some centers wanting to adopt SSRF may struggle with indication for operative management and identification of patients who would most benefit from the surgery [7].

Barriers may also include surgeon comfort with the technique as surgeons do not routinely use internal fixation equipment and tools. Additionally, this is not a requirement for surgical residency. To overcome this barrier, our level 1 trauma center developed a tandem operative approach utilizing orthopedic and trauma surgeons. We sought to investigate outcomes in the SSRF patients and studied the benefit of a tandem approach. Our *hypothesis* stated, patients who underwent the SSRF would have significantly better outcomes than nonoperative controls in terms of survival, ICU duration, and rates of pneumonia (VAP).

## 2 Methods

### 2.1 SSRF program

In this single institution, SSRF was performed from 2015 to 2017, but due to staff turnover there was a gap in availability of SSRF. The program was restarted in 2018 but there was a lack of experienced staff regarding the SSRF approach particularly with high-risk patients. To counter this, we utilized a multidisciplinary tandem operative approach, allowing us to leverage talent and immediately take on difficult cases. Our experience may offer a unique perspective or a possible solution to the problem of adoption.

*Tandem, multidisciplinary approach***.** At the time we developed our rib fracture program, we had trauma patients who met the indications for stabilization, but our trauma surgeons had limited experience with placement of the hardware (plates and screws) required for the procedure. We asked for assistance from the orthopedic surgeon who had interest and experience with this type of hardware. Although we started the practice to gain familiarity with this portion of the technique, we continued it for convenience. The trauma surgeon identified the case and coordinates scheduling with the orthopedic surgeon. The trauma surgeon begins the case by identifying the optimal patient position based on three dimensional reconstructions of the injured chest wall on computed tomography scanning. The trauma surgeon also provides operative exposure with the incision and placement of a thoracostomy tube. The orthopedic surgeon then is called in, places the hardware, and the trauma surgeon closes the incisions.

There is significant variability from center to center and within each center regarding indications Our study indications were: Flail chest with >3 segments; non-flail with severe, bi-cortical displacement of >3 contiguous segments. Secondary considerations were intubation for respiratory failure due to fractures, chest wall deformity with estimated loss of volume >10%, failure of medical management (oral analgesia and regional pain catheter), inability to work with physical & occupational therapy secondary to unstable chest wall or uncontrolled pain, hypoxemic respiratory failure thought to be secondary to fractures, physical exam finding of unstable chest wall by palpation.

If a patient had comorbidity there was special consideration and evaluation by echocardiogram. Regarding Relative Contraindications, we noted apical fractures in segments 1–3, only 1–2 contiguous severe fractures, and unable to tolerate a general anesthesia or moderate risk thoracic procedure due to co-morbidity or associated injuries. Patients were all evaluated after a 24-h period of optimal medical therapy using physical therapy evaluation, bedside incentive spirometry, and subjective pain scores. Adjunctive evaluation included chest x-ray, arterial blood gas and vital signs-specifically oxygen saturation by pulse oximetry.

Trauma patients with rib fractures who met criteria for admission were admitted to either the ICU or hospital ward. 3d reconstructions of chest CT was performed in any patient with multiple rib fractures. These images were viewed and discussed collaboratively between trauma and orthopedic teams. Each patient was evaluated for hypoxia, pain, incentive spirometry totals and performance with physical therapy. Comorbidities, overall injury burden, timing of other procedures and cardiovascular fitness were also taken into consideration. Selected patients based on age or uncontrolled pain were offered regional anesthesia, which in our center is erector spinae plane (ESP) anesthesia with an OnQ system. Patients with flail segments or >3 rib fractures with severe bi-cortical displacement (non-flail) indications were offered surgery, when clinically appropriate. Chest surgery was performed in muscle-sparing fashion and included evacuation of hemothorax when clinically appropriate. A chest tube was placed in the pleural space. If the patient had not already received regional anesthesia, an OnQ catheter was placed under direct vision on the chest wall posterior to the injured segments.

This retrospective study investigated the impact of operative fixation in patients. After institutional review board approval was obtained (IRB # 1771804-1), SSRF patients, enrolled in the trauma registry from January 2018 to January 2021, were reviewed retrospectively. Demographics, medical comorbidities, mechanism of injury, flail diagnosis, operative characteristics, hospital and ICU length of stay (LOS), ventilator days, ventilator-associated pneumonia VAP, tracheostomy, mortality rates, abbreviated Injury Score (AIS), Injury Severity Score (AIS ISS), and Glasgow coma scale (GCS) data were collected.

### 2.2 Group matching criteria

Data extracted from the institutional SSRF database were combined with the dataset on nonoperative controls for *case control matching* with our statistical analysis software (SPSS version 28.0, IBM Corp) so that comparative analysis between SSRF patients and nonoperative controls could be performed. Case controls were selected with the SPSS software using the following five criteria: Age, Abbreviated Injury Scale (AIS), Injury Severity Score (ISS), Glasgow Coma Scale (GCS), and sex.

### 2.3 Statistical analysis

Demographic and clinical characteristics were summarized using descriptive statistics. Descriptive analyses were conducted by group patient characteristics (age, sex), injury characteristics (number of rib fractures, AIS for thorax body region, and Injury Severity Score (ISS) and outcomes (total hospital days LOS, total intensive care unit (ICU) days, total ventilator days, and in-hospital mortality). The pulmonary complication evaluated was ventilator-associated pneumonia (VAP), a concern in this healthcare system. X2 or Fisher's exact test was used for categorical variables. Group comparisons of proportions were made using Chi-square tests or Fishers exact tests where numbers were small and were reported as numbers (%). All variables were assessed for normality.

Independent measures *t*-tests for LOS were employed. Student's t-test or Wilcoxon-Mann-Whitney test was used for continuous variables. All categorical variables were described as numbers and percentages. All continuous variables were described as means with standard deviation (SD). All p values are 2-sided, with a critical significance level of ≤0.05. Data analysis was performed using SPSS version 26.0 (IBM Corp).

## 3 Results

This retrospective study had a total of 86 patients including 43 patients who underwent a multidisciplinary operative approach to rib-plating (SSRF) from January 2018 to January 2021 and 43 non-operative controls matched on demographics and clinical data. SSRF was performed at a median of three days. The nonoperative equivalent controls were patients with the same disorder who did not undergo SSRF.

### 3.1 General clinical data of SSRF and nonoperative groups

No significant differences between groups emerged regarding the five matching variables, including age (*p* = 0.66), AIS (*p* = 0.68), AIS ISS (*p* = 0.34) or GCS (*p* = 0.08). Chi-square tests found no difference between patient groups (SSRF and nonoperative) regarding sex. There were 31 males in each group, (Table 1).

**Table 1:** Patient demographics SSRF (operative) and non-operative (non-operative) groups.

Subgroups	SSRF group (n = 43)	Non-Operative (n = 43)	p Value
Age, y, mean SD	59 (17.34)	57.34 (17.88)	0.66
AIS code mean, SD	3.00 (.64)	3.00 (.412)	0.68
AIS ISS mean SD	19.6 (9.8)	21.1 (11.8)	0.34
GCS mean, SD	13.4 (3.70)	12.1 (5.06)	0.08
Sex male/female	31/12	31/12	1.00

•AIS, Abbreviated Injury Scale; The Injury Severity Score (ISS); SD = Standard Deviation; NS= No statistically significant difference between groups; GCS, Glasgow Coma Scale.

### 3.2 Patient injury description results

There were no significant differences between groups in the number of ribs fractured (*p =* 0.09), flail chest *(p* = 0.78) or the degree of underlying lung contusion. (*p* = 0.58). Similarly, there was no difference between groups regarding mechanism of injury (*p* = 0.65). The majority of injuries were caused by a fall from height a motor-cycle crash and a motor vehicle accident. Less injuries were caused by ground level falls (GLF). There was a statistically significant difference in those with pneumothorax (*p* = 0.02). See Table 2.

**Table 2:** Injury description: SSRF (operative) and non-operative groups.

Subgroups	SSRF group (n-43)	Non-Operative (n = 43)	p Value
Number of fractured ribs (%)	7.7 (18%)	6.0 (14%)	0.09
Flail chest (%)	8 (18.6%)	7 (16%)	0.78
Pneumothorax, n (%)	32 (74%)	25 (57%)	0.02
Contusion n, (%)	20 (46.5%)	17 (39%)	0.58
Mechanism of injury, n (%)			0.652
Fall from height	14 (33%)	5 (12%)	
Motor-cycle crash	13 (30%)	13 (30%)	
Motor vehicle accident	11 (25%)	16 (37%)	
Ground level fall (GLF)	3 (7%)	6 (14%)	
Crush injury	2 (5%)	3 (7%)	
Other surgical interventions, n (%)	12 (28%)	11 (26%)	
Thoracotomy, n	1	0	
Laparotomy, n	1	1	
Orbital wall, n	1	0	
ORIF clavicle, n	3	0	
ORIF (other)*, n	3	6	
Lung cancer, n	1	0	
Thoracic fusion, n	1	1	
Plastics, n	1	0	
Hand surgery	0	1	
Craniotomy	0	2	

• Includes ORIF, Open reduction internal fixation; ORIF other* includes femur, humerus, orbital floor, pelvic, right acetabulum.

### 3.3 Primary outcomes

Results of SSRF included significant decreases in mortality (*p* = 0.03) and in ICU duration. Patients with SSRF had a significantly shorter duration in the ICU than the nonoperative group (*p* = 0.01). Less patients in the SSRF group developed ventilator-associated pneumonia but the difference was not significant (Table 3).

**Table 3:** Outcomes: SSRF (operative) and non-operative (NonOp) group comparison with acute phase of index.

Outcomes	SSRF group (n = 43)	NonOp group (n = 43)	p Value
**ICU stay mean (day/SD)**	**8.72 (7.95)**	**14.28 (9.06)**	**0.01**
**Hospital stay mean (day/SD)**	**12.81 (9.14)**	**14.93 (11.51)**	**0.35**
**In hospital mortality, n (%)**	**1 (2%)**	**7 (16%)**	**0.03**
**Pneumonia VAP, n (%)**	**1 (2%)**	**6 (14%)**	**0.06**
**MV time, mean (day/SD)**	**8.22 (8.34)**	**9.24 (6.5)**	**0.59**

•*SD* = Standard Deviation; VAP = Ventilator-Associated pneumonia; MV time = Mechanical Ventilator time (days).

## 4 Discussion

While the number of patients with rib fractures receiving SSRF has increased, the number of patients undergoing this treatment remains small [22,23]. Not every trauma center provides SSRF and some struggle with the indication for operative management and identification of patients who will most likely benefit from fixation [7,11]. One other barrier to SSRF may include surgeon comfort with the rib fixation technique as trauma surgeons do not routinely use internal fixation equipment and tools. We confronted this issue by adding an orthopedic surgeon to the trauma surgery team. This current retrospective study investigated the impact and benefits of SSRF patients compared with non-operative equivalent controls.

The results of this study and recent investigations are encouraging as the reported data reveal general decreases in complications [[6], [7], [8],^10^]. Consistent with the literature, our study found surgical fixation of rib fractures significantly decreased mortality compared with nonoperatively managed controls [7,8]. Additionally, our SSRF study showed reduction in intensive care unit duration (ICU), supporting prior literature [8,21]. Swart and colleagues [8] concluded SSRF decreased mortality, ICU time, and pulmonary complications. Despite the two significant findings concerning decreased mortality and ICU time, our investigation found no significant differences regarding VAP, a great concern in this healthcare system. However, similar to findings by Marasco [21], there was a trend toward more patients in the nonperative group developing pneumonia (*p* = 0.06; Table 3).

Historically, nonoperative management has been the standard of care and may have been influenced by a knowledge gap [8]. Surgical fixation of rib fractures has gained significant momentum but is still relatively a new technique. Each trauma center faces its own unique set of challenges to adoption of SSRF. Local credentialing, past experience and training, interest level and available personnel may all be local factors that dictate which specialty performs SSRF within each trauma center. Our team approach between orthopedic surgery and trauma surgery worked well and immediately allowed us to take on a number of high-risk cases of varying complexity level safely, with minimal need for transfer.

There is an area where a future study could expand these findings. Further investigations could expand to include the SSRF team approach in a multi-institutional study. Additionally evaluating the cost-effectiveness of rib plating outcomes is warranted. However, our data would suggest likely cost savings, particularly with length of stay in the ICU.

Limitations of this study include a small sample size and retrospective nature of data collection with all procedures performed at a single institution. Retrospective studies can suffer from selection bias; however, we used measures to counter this bias. Also, many patients admitted with polytrauma have multiple injury diagnosis and procedure code, thus challenging direct connections between a single procedure and outcomes. AIS was used during case matching in order to match injury pattern; however, we acknowledge that AIS does not match fracture pathology and may add additionally to selection bias. All operative patients received regional anesthesia, whereas the nonoperative group often did not, which may affect the results of the study. Finally, since many of our patients discharged prior to 30 days and many did not follow up in clinic, we may have missed long-term outcomes.

As noted in the literature, surgeries should be conducted by surgeons or a combined surgical team comfortable with both thoracic anatomy and exposure and with internal rib fixation techniques [8]. To our knowledge, the SSRF tandem approach is unique to our level 1 trauma center. Findings were very compelling for SSRF and for the tandem, team operative approach. The orthopedic and trauma team strategy had many benefits as it was able to leverage skills and expertise and increase collaboration between services. Moreover, we were able to complete more difficult reconstructions. In our population of blunt poly-trauma patients with moderate to severe injuries, we report improved survival and significant decreases in the length of ICU stay. The team solution may allow for wider adoption of SSRF in the appropriately selected trauma patient. The SSRF team gained momentum by taking on a larger volume of moderate-to severely-injured-patients.

The significant clinical benefit in the selected patient with rib fractures suggests strong consideration should be given to operative intervention by a multidisciplinary effort. Our experience may serve as a model for trauma centers that may be interested in starting a new program or enhancing current service offerings.

## Author contribution statement

Frank Bauer: Conceived and designed the experiments; Performed the experiments; Analyzed and interpreted the data; Wrote the paper.

Susan Haag: Conceived and designed the experiments; Analyzed and interpreted the data; Contributed reagents, materials, analysis tools or data; Wrote the paper.

Kaveh Najafi; Brian Miller; John Kepros: Performed the experiments; Wrote the paper.

## Data availability statement

Data included in article/supplementary material/referenced in article.

## Declaration of interest's statement

The authors declare no conflict of interest.
